# ^18^F‐PSMA‐1007 PET/CT Demonstrates Higher Accuracy for Biochemically Recurrent Prostate Cancer Compared to Contemporary Conventional Imaging

**DOI:** 10.1155/aiu/8550174

**Published:** 2026-07-05

**Authors:** Zachary Stanton, Bonnie Woloschuk, Alexander Tamm, Ryan Hung, Adwait Trivedi, Michael Kolinsky, Nawaid Usmani, Lindsay Rowe, Ronald Moore, Frank Wuest, Jonathan Abele

**Affiliations:** ^1^ Department of Radiology and Diagnostic Imaging, University of Alberta, Edmonton, Alberta, Canada, ualberta.ca; ^2^ Department of Oncology, University of Alberta, Edmonton, Alberta, Canada, ualberta.ca; ^3^ Department of Surgery, University of Alberta, Edmonton, Alberta, Canada, ualberta.ca

**Keywords:** biochemical recurrence, bone scan, CT, PET/CT, prostate cancer, PSMA-1007

## Abstract

**Purpose:**

The goal of this study was to compare the accuracy of ^18^F‐PSMA‐1007 PET/CT to contemporary conventional imaging (contrast‐enhanced CT scan of the chest, abdomen, and pelvis and a ^99m^Tc‐MDP whole body bone scan with SPECT/CT of the torso) in the setting of biochemically recurrent prostate cancer.

**Methods:**

This prospective nonrandomized Phase II cohort study clinical trial included 85 patients with biochemically recurrent prostate cancer (previous therapy). ^18^F‐PSMA‐1007 whole body PET/CT imaging was performed in all patients, with conventional imaging (contrast‐enhanced CT scan of the chest, abdomen, and pelvis and a ^99m^Tc‐MDP whole body bone scan with SPECT/CT of the torso) then performed within 12 days. PROMISE criteria were used for PET/CT interpretation. After a minimum 1‐year follow‐up period, lesion‐level and patient‐level analyses were performed based on previously published reference criteria (Lawhn‐Heath et al. 2019). The accuracy data were compared. Referring physicians were surveyed to help determine if the scans altered treatment. Categorical variables were compared using the McNemar test (*p* < 0.05 significant).

**Results:**

53 patients with a total of 72 evaluable lesions were included in the analysis. ^18^F‐PSMA‐1007 PET/CT had a higher sensitivity than conventional imaging on a lesion level (100% compared with 58.3%) and higher overall accuracy on a patient level (90.6% compared with 62.3%, *p* < 0.05). The referring physicians felt that the ^18^F‐PSMA‐1007 PET/CT scans were beneficial to the clinical care of the patient in 90.6% of cases.

**Conclusion:**

Overall, the findings support the use of ^18^F‐PSMA‐1007 over conventional imaging in the evaluation of patients with biochemically recurrent prostate cancer.

**Trial Registration:** ClinicalTrials.gov.identifier: NCT04733768

## 1. Introduction

Prostate cancer is the most common cancer to affect Canadian men (excluding nonmelanoma skin cancers), with an estimate of 27,900 new cases in 2024. Despite an overall 5‐year survival rate of 91%, the high incidence results in prostate cancer being the third leading cause of cancer deaths in Canadian men [1]. Ongoing developments in the field of nuclear medicine are promising for the early detection and treatment of prostate cancer, with novel diagnostic positron emission tomography (PET) tracers a key area of study [2, 3].

(((S)‐1‐carboxy‐5‐((S)‐2‐(4‐(((S)‐4‐carboxy‐2‐((S)‐4‐carboxy‐2‐(6‐[18F]fluoronicotinamido)butanamido)butanamido)‐methyl)benzamido)‐3‐(naphthalen‐ 2‐yl)propanamido)pentyl)carbamoyl)‐L‐glutamic acid (^18^F–prostate‐specific membrane antigen [PSMA]–1007) is a Glu–urea–based compound with high affinity for PSMA [4]. PSMA is a Type II transmembrane protein which demonstrates increased ductal luminal expression in the setting of prostate cancer [5]. PSMA PET imaging is an established technique worldwide with a variety of ^68^ Ga and ^18^F‐based tracers utilized, all demonstrating a higher accuracy in the evaluation of prostate cancer than previous computed tomography (CT) and nuclear medicine imaging techniques [6]. ^18^F‐PSMA‐1007 is a second‐generation PSMA PET tracer with potential advantages over other tracers, including a high PSMA affinity, rapid background clearance, higher spatial resolution than ^68^ Ga, and longer half‐life (110 min) than ^68^ Ga (68 min), resulting in potentially greater geographic portability and clinical flexibility [7]. Importantly, in comparison to other ^18^F‐PSMA PET tracers such as ^18^F‐DCFPyL, ^18^F‐PSMA‐1007 demonstrates less urinary excretion, potentially improving the assessment of the pelvic region (i.e., less background activity).

In general, PSMA PET imaging appears to be of a diagnostic benefit in patients with biochemically recurrent (BCR) prostate cancer after definitive therapy (radical prostatectomy, external beam radiotherapy, cryotherapy, or brachytherapy) based on elevated serum prostate‐specific antigen (PSA) levels and negative conventional imaging (contrast‐enhanced CT scan of the chest, abdomen, and pelvis as well as a ^99m^Tc‐MDP bone scan) [8, 9]. In particular, PSMA PET appears to have much higher sensitivity than conventional imaging at low PSA levels (0.2–0.5 μg/L) [10].

The purpose of this study was to compare the accuracy of ^18^F‐PSMA‐1007 PET/CT with contemporary conventional imaging (within 12 days) in patients with BCR prostate cancer at both an individual lesion level and at a patient level. We also aimed to assess the impact of ^18^F‐PSMA‐1007 PET/CT imaging on treatment decisions for this patient group.

## 2. Methods

### 2.1. Inclusion/Exclusion

This single‐center prospective nonrandomized Phase II cohort study was approved by Health Canada as well as our local institutional ethics review board (HREBA.CC‐20–0281) and was registered prospectively with ClinicalTrials.gov. Informed consent was obtained from all participants.

This study included 85 participants with BCR prostate cancer who met one of the following inclusion criteria:

1. Adult patient (≥ 18 years old) with a history of radical prostatectomy for the treatment of prostate cancer and a serum PSA > 0.2 μg/L
2. Adult patient (≥ 18 years old) with a history of radiotherapy, cryotherapy, or brachytherapy for the treatment of prostate cancer and a serum PSA progressively rising to ≥ 2 μg/L (minimum two samples), or a serum PSA doubling‐time of < 9 months

Participants were enrolled based on referrals from regional physicians who deemed the imaging studies potentially useful for clinical care.

Exclusion criteria for the study included the following: inability to obtain consent, weight greater than 225 kg (weight limitation of PET/CT scanner), inability to lie flat for 30 min to complete the PET/CT imaging session, lack of intravenous access, having already had both CT scan of the chest, abdomen, and pelvis and ^99m^Tc‐MDP bone scan within 3 months prior, or a history of allergic reaction to ^18^F‐PSMA‐1007 or ^99m^Tc‐MDP.

### 2.2. Study Group

All participants had an^18^F‐PSMA‐1007 PET/CT scan as well as conventional imaging (including an IV contrast‐enhanced CT scan of the chest, abdomen, and pelvis as well as a ^99m^Tc‐MDP whole body bone scan with SPECT/CT of the torso) within 12 days of each other. Clinical chart data were reviewed a minimum of 1 year after the scan to assess lesion positivity based on previously published criteria (11, Supporting File).

Eighty‐five participants met the inclusion criteria and were enrolled in the study. Two [2] participants died prior to follow‐up and were excluded from analysis. One [1] participant did not follow the study protocol (no bone scan with SPECT/CT within 12 days of the ^18^F‐PSMA‐1007 PET/CT scan). Twenty‐nine participants were excluded from analysis as they did not satisfy the reference standard criteria (typically, a lack of follow‐up histopathology and imaging). Fifty‐three participants were included in the analysis (Figure 1).

**FIGURE 1:**
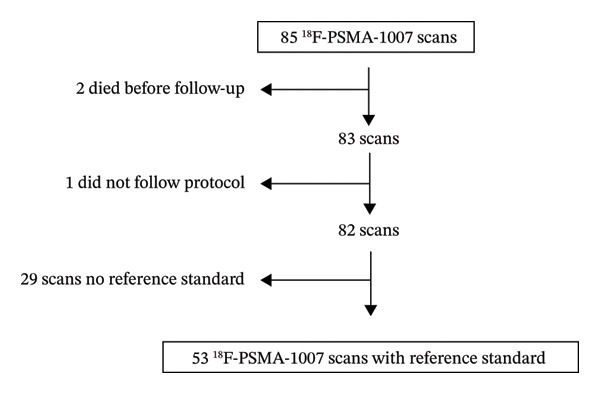
Flowchart demonstrating study exclusions resulting in the final study population of 53 patients/scans.

In assessing the estimated required sample size prior to the study, the following conservative estimates were used: lesion prevalence 60%, sensitivity conventional 50%, sensitivity test 70%, *α* of 0.05, and power of 0.80. Based on this, a minimum sample size of 82 was required in our initial study plan [11].

^18^F‐PSMA‐PET/CT scan technique.

^18^F‐PSMA‐1007 was performed according to the following parameters:

4 MBq/kg (max 400 MBq) of ^18^F‐PSMA‐1007 (Edmonton Radiopharmaceutical Center, Edmonton, Alberta) was injected intravenously with the patient reclined in a standard uptake room. After a 2 h uptake time from injection (mean: 124 ± 22 min; range: 78–234 min), participants were transferred to the PET scanner table (GE Discovery MI PET/CT, GE Healthcare Canada Inc, Mississauga, Ontario). The participants were asked to void completely immediately prior to scanning. Whole‐body (top of skull to toes) PET acquisition was acquired at 2 min per bed position. A CT scan of the same anatomic region was acquired without the administration of intravenous iodinated contrast or oral contrast. CT acquisition parameters included the following: 120 kv, 300 mAs, 3.75 mm helical slice thickness, and FOV 50–70 cm.

### 2.3. Conventional Imaging Technique

All patients had a comparison conventional imaging study performed within 12 days of the investigational PET/CT scan.

Serum creatinine was measured within 10 days prior to the scan for all patients, with eGFR calculated.

A ^99m^Tc‐MDP whole body planar scan with SPECT/CT scan of the torso was performed in all patients. 1000 MBq (range: 900–1110 MBq) of ^99m^Tc‐MDP was injected intravenously. After a delay of 3‐4 h (uptake time), the participant was transferred to the SPECT/CT scanner table (GE Discovery 670 16‐slice SPECT/CT system, GE HealthCare Canada Inc., Mississauga, Ontario). Initially, whole body planar images were acquired (arms down, scan speed 10 cm/min, low‐energy high‐resolution collimator, matrix size 512 × 1024). After reviewing these images, specific static planar images in different projections may have been obtained at the discretion of the involved nuclear medicine physician. Subsequently, SPECT was performed using two 40 cm FOV acquisitions with 4 cm overlap extending from the clavicles to the bottom of the pelvis. For individuals with a torso > 76 cm in length, images were acquired from the bottom of the pelvis up. SPECT acquisition parameters included 120 projections (3° angular sampling), 20 s/projection, total SPECT imaging time 40 min, and 128 × 128 matrix. A low‐dose noncontrast‐enhanced CT scan of the same region was obtained for attenuation correction (120 Kv, 100 mAs, free breathing).

For all participants included in the analysis, eGFR was ≥ 40 mL/min/1.73 m^2,^ and there was no history of contrast allergy. An IV contrast‐enhanced CT was performed in all participants using the same SPECT/CT scanner. This included two initial scout images for range planning. 100 mL of Omnipaque 350 (GE HealthCare Canada Inc., Mississauga, Ontario) was injected intravenously at a rate of 3.5 mL/s, followed by 25 mL of normal saline at a rate of 3 mL/s. At 25 s postinjection, helical CT images were obtained from the clavicles to the bottom of the diaphragm (120 kV, 250 mAs). At 80 s postinjection, helical CT images were obtained from the top of the diaphragm to the lesser trochanters of the proximal femurs (120 kV, 300 mAs). Parameters for the helical CT acquisitions include 50–70 cm FOV, 1.375:1 pitch, 0.8 s rotation speed, and helical slice thickness 3.75 mm. End‐inspiration breath‐hold was used. No oral contrast was administered.

### 2.4. Lesion Analysis

All PET/CT scans were reviewed using Oasis workstation software (Segami Corporation, Laval, Quebec). In all cases, the ^18^F‐PSMA‐1007 PET/CT scan was reviewed first, followed by review of the conventional imaging scans. Any identified abnormal lesion on either the ^18^F‐PSMA‐1007 PET/CT scan or the conventional imaging scans was directly compared and categorized as follows:

Category A: the lesion was identified on ^18^F‐PSMA‐1007 PET/CT but not on the conventional imaging scans.

Category B: the lesion was identified on both the ^18^F‐PSMA‐1007 PET/CT as well as the conventional imaging scans.

Category C: the lesion was identified on the conventional imaging, but not on the ^18^F‐PSMA‐1007 PET/CT scan.

With respect to ^18^F‐PSMA‐1007 PET/CT interpretation, bone lesions were characterized using the criteria defined by Arnfeld et al. [12], and soft tissue lesions were characterized using criteria from the PROMISE study [13].

### 2.5. Reference Standard Criteria

Clinical data were reviewed at a minimum of 1 year after the scans to define reference positivity on a lesional and patient level.

Lesional histopathology results were used as the reference standard when available.

When histopathology was unavailable, lesional positivity was determined based on previously published criteria [14].

If these criteria for determining lesion positivity were not met, the participant data were excluded from the analysis.

For the patient‐level analysis, a participant with at least a single reference positive lesion was considered positive. If there were no follow‐up imaging studies with evidence of positive disease, no histopathology results identifying positive disease, and the PSA decreased by greater than 50%, the participant was considered negative in follow‐up. If the participant did not meet these criteria and did not meet the lesional reference standard criteria, the participant was excluded from the patient‐level analysis.

### 2.6. Referring Physician Survey

Referring physicians were contacted after the scan to complete a postscan survey (minimum 6 months after the scan). The survey asked if they felt that the ^18^F‐PSMA‐1007 PET/CT scan was beneficial to the clinical care of the patient (yes or no). If yes, the perceived reason for the benefit was collected and categorized as follows: changed surgical approach, changed radiotherapy approach, changed from surgery to radiotherapy, changed from radiotherapy to surgery, changed from radiotherapy/surgery to medical therapy, changed from medical therapy to radiotherapy/surgery, changed to palliative therapy, or other.

### 2.7. Statistical Analysis

Categorical statistics were compared using a McNemar test. 95% confidence intervals for categorical variables were calculated using an online calculator (omnicalculator.com/statistics/confidence interval). Continuous variables are presented as mean ± standard deviation. *p* values < 0.05 were considered significant.

## 3. Results

### 3.1. Patient Demographics

Fifty‐three participants were included in the analysis (Table 1).

**TABLE 1:** 53 included participants information.

	**Mean ± SD**	**Range**	**Units**

Age	66.5 ± 7.0	51–79	years
Serum PSA	4.9 ± 7.3	0.2–32.4	μg/L
Time between imaging studies	3.4 ± 2.9	1–12	days
Time from PET/CT to follow‐up	19.0 ± 1.9	16–23	months

Abbreviations: PET/CT = positron emission tomography/computed tomography; PSA = prostate‐specific antigen; SD = standard deviation.

### 3.2. Per Patient Analysis

On a patient level, ^18^F‐PSMA‐1007 PET/CT demonstrated 33 true positive (TP) studies, 0 false positive (FP) studies, 15 true negative (TN) studies, and 5 false negative (FN) studies. This resulted in a sensitivity of 86.8% (95% CI: 77.7–95.9), specificity of 100%, positive predictive value (PPV) of 100%, and negative predictive value (NPV) of 75% (95% CI: 63.3–86.7). The overall accuracy was 90.6% (95% CI: 82.7–98.4).

Conventional imaging demonstrated 18 TP, 0 FP, 15 TN, and 20 FN. This resulted in a sensitivity of 47.4% (95%CI 40.0–60.8), specificity of 100%, PPV of 100%, and NPV of 42.9% (95% CI: 29.6–56.2). The overall accuracy was 62.3%.

In patients with disease, ^18^F‐PSMA‐1007 PET/CT was significantly more accurate than conventional imaging (*p* < 0.05).

### 3.3. Per Lesion Analysis

A total of 72 evaluable lesions were analyzed. All lesions were identified in the prostate bed, lymph nodes, or bones. There were no metastatic lesions identified outside of these regions on either the ^18^F‐PSMA PET/CT imaging or the conventional imaging studies.

These lesions included 10 lesions (14%) within the prostate bed, 32 lesions (44%) involving lymph nodes, and 30 lesions (42%) involving bone.

Category A (lesions identified on ^18^F‐PSMA‐1007 PET/CT but not on conventional imaging) included 30 lesions (42%). Category B (lesions identified on both ^18^F‐PSMA PET/CT and conventional imaging) included 42 lesions (58%). There were no Category C lesions.

With respect to the prostate bed, all lesions (10/10, 100%) were Category A.

With respect to lymph nodes, 18/32 (56.3%) were Category A and 14/32 (43.8%) were Category B.

With respect to bone lesions, 2/30 (6.7%) were Category A and 28/30 (93.3%) were Category B.

As lesions were only identified based on imaging, the number of TN lesions cannot be reliably assessed (absent lesions on both studies cannot be quantified). The FN rate can be estimated based on the identification of a lesion on at least 1 scan type.

Based on this, on a per lesion level, ^18^F‐PSMA‐1007 PET/CT demonstrated 72 TP, 0 FP, 0 FN, a sensitivity of 100%, and a PPV of 100%. Conventional imaging demonstrated 42 TP, 0 FP, 30 FN, a sensitivity of 58.3% (95% CI: 47.0–69.7), and a PPV of 100% (Figure 2).

**FIGURE 2:**
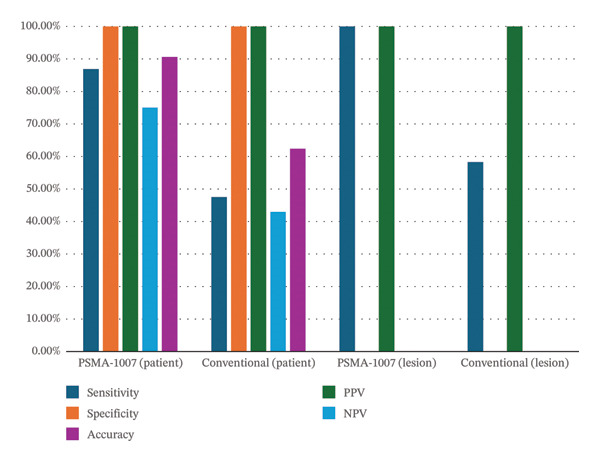
Histogram demonstrating accuracy statistics comparing ^18^F‐PSMA‐1007 PET/CT and conventional imaging (CT C/A/*p* and whole body ^99m^Tc‐MDP bone scan with SPECT/CT of the torso) on a per‐patient and per‐lesion basis.

In this context, ^18^F‐PSMA‐1007 has a significantly higher sensitivity for recurrent disease than conventional imaging (*p* < 0.05).

### 3.4. Referring Physician Survey

The survey completion rate was 100%. The mean time from ^18^F‐PSMA‐1007 PET/CT scan to survey completion was 209 ± 40 days (range: 164–367 days).

For the question of whether the referring physician felt that the ^18^F‐PSMA‐1007 PET/CT scan was beneficial to the clinical care of the patient, 48/53 (90.6%) answered “Yes” and 5/53 (9.4%) answered “No.”

Specific reasons for perceived benefit to clinical care included the following: changed surgical approach (3/53, 5.7%), changed radiotherapy approach (15/53, 28.3%), changed from surgery to radiotherapy (0/53, 0%), changed from radiotherapy to surgery (0/53, 0%), changed from radiotherapy/surgery to medical therapy (10/53, 18.9%), changed from medical therapy to radiotherapy/surgery (7/53, 13.2%), changed to palliative therapy (2/53, 3.8%), and other (11/53, 20.8%) (Figure 3).

**FIGURE 3:**
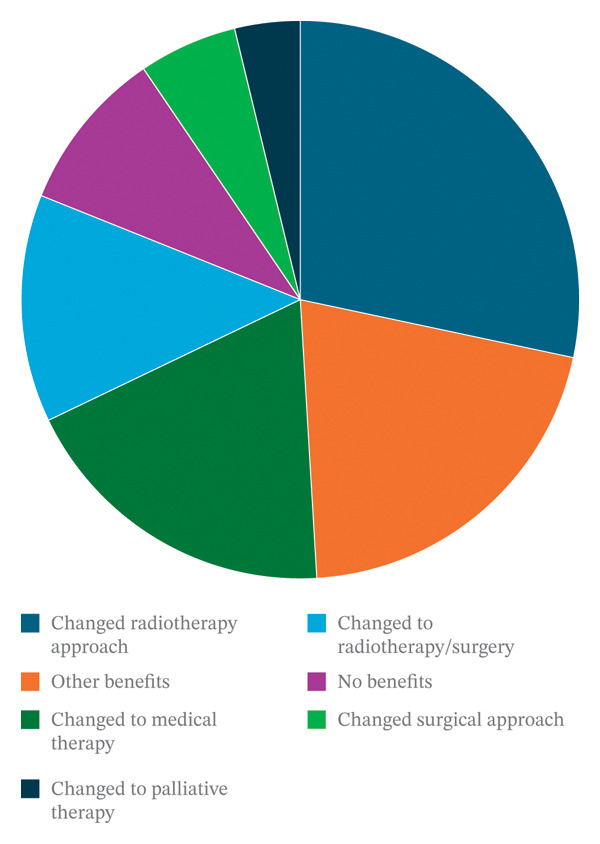
Pie chart demonstrating relative proportions of reasons for perceived clinical benefit of ^18^F‐PSMA‐1007 PET/CT based on referring physician survey.

Other reasons documented included the following: changed from active surveillance to antiandrogen therapy, no evidence of recurrent disease, confirmed local disease for salvage cryoablation after radiotherapy, change in medical therapy, intensification of medical therapy, biopsy‐proven cervical lymph nodes, allowed continued observation, changed from radiotherapy to medical, and informed decision to proceed with salvage radiotherapy.

For surveys that answered “No,” documented reasons included the following: did not change management, PET identified more bone and lymph node metastases but did not change therapy, no site of recurrence identified, diffuse metastases also noted on conventional imaging, and no change in therapy.

## 4. Discussion

The results of this study confirm that ^18^F‐PSMA‐1007 PET/CT has a high overall sensitivity for the detection of recurrent prostate cancer, with greater sensitivity and accuracy compared to conventional imaging, particularly for prostate bed lesions (Figure 4) and lymph node metastases (Figure 5). On a per patient level, the overall accuracy of ^18^F‐PSMA‐1007 PET/CT was 90.6% compared to 62.3% for conventional imaging.

**FIGURE 4:**
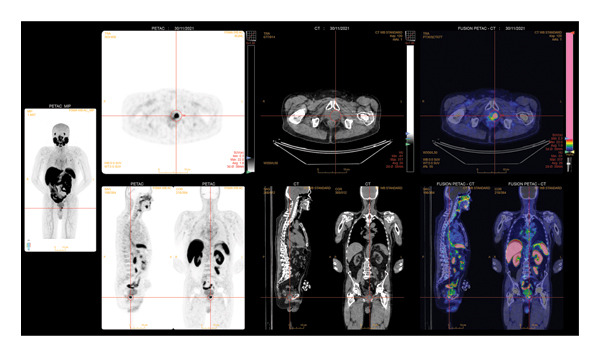
Example of an ^18^F‐PSMA‐1007 PET/CT scan demonstrating recurrence in the prostate bed. This patient was treated with external beam radiation for prostate cancer 3 years prior with curative intent. PSA had risen to 2.58 μg/L 2 months prior to the scan. Intense activity at the left prostate gland (crosshair) was compatible with recurrence. Subsequent biopsy was positive for recurrent tumor at the left gland (all left‐sided biopsies positive; all right‐sided biopsies negative). The patient underwent salvage whole gland cryoablation with subsequent undetectable PSA.

**FIGURE 5:**
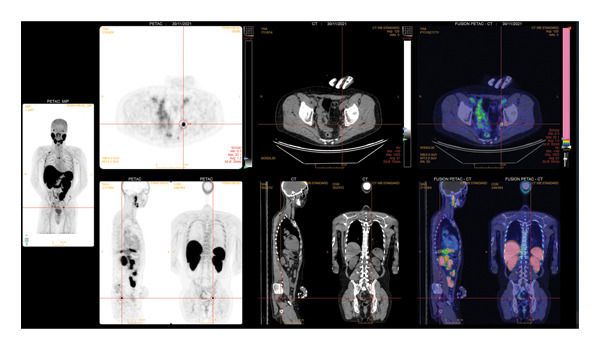
Example of an ^18^F‐PSMA‐1007 PET/CT scan demonstrating recurrence involving a left internal iliac lymph node. This patient was treated with radical prostatectomy and bilateral lymph node dissection 2 years prior. An initial postoperative PSA elevation to 0.2 μg/L 6 months later was treated with salvage radiotherapy to the prostate bed. The PSA reached a nadir of 0.1 μg/L but then rose to 0.39 μg/L 1 year prior to the scan. Intense activity (greater than liver) is demonstrated involving a 6‐mm diameter left internal iliac lymph node (crosshair) compatible with recurrence.

Our results are similar to multiple previous studies demonstrating a high sensitivity for PSMA PET in the setting of BCR. Hope et al. performed a meta‐analysis concluding that ^68^Ga‐PSMA‐11 PET had a detection rate of 94% with PSA > 2.0 and a PPV of 99% [10]. A more recent review by Armany et al. summarized studies specifically evaluating ^18^F‐PSMA‐1007 PET/CT in the setting of BCR [15]. A reported sensitivity of 95% was noted, with a higher lesion detection rate associated with higher PSA values. Similar to our study, the reported accuracy was high for prostate bed lesions and lymph node metastases, but relatively lower for bone lesions due to reduced specificity for ^18^F‐PSMA‐1007 uptake within bone.

A recent overview of reviews by Dullea et al. evaluating the diagnostic accuracy of ^18^F‐PSMA PET/CT radiotracers in patients with biochemical recurrence concluded that the current evidence base is broadly supportive of ^18^F‐PSMA PET/CT imaging in re‐staging this patient group [16].

Our study strengthens this assessment by directly comparing ^18^F‐PSMA‐1007 PET/CT with contemporary conventional imaging, including an IV contrast‐enhanced CT scan of the chest, abdomen, and pelvis, along with a ^99m^Tc‐MDP bone scan with both whole body planar and torso SPECT/CT components, performed within 12 days of each other in a prospective fashion in all patients. This eliminates concerns around temporal bias and lesion progression between studies interfering with scan comparisons. As well, our bone scan protocol, including SPECT/CT of the torso in all participants, is reflective of modern high‐quality imaging practice in this population, not reflected in many previous comparative studies (typically planar bone scan imaging data only).

We attempted to measure the clinical impact of performing an ^18^F‐PSMA‐1007 PET/CT scan in this population through a referring physician survey. Clinical reports for both the ^18^F‐PSMA‐1007 PET/CT scan as well as the conventional imaging were available to these physicians and their patients. The referring physicians felt the results of the PET/CT scans were beneficial to the clinical care of their patients in 90.6% of cases, further supporting the use of ^18^F‐PSMA‐1007 PET/CT imaging in this patient group.

One limitation of the study is the high number of lesions with no available reference standard. Of 82 scanned patients, data from only 53 patients were included in the analysis, as lesion data from the other 29 patients did not meet the criteria for reference standard evaluation. This exclusion was mainly due to a lack of histopathology and a lack of follow‐up imaging despite a relatively long follow‐up window (range: 16–23 months). As well, nearly all patients received systemic androgen deprivation as the standard of care, limiting the utility of local therapy only criteria.

Another limitation of the study is the low number of negative scans potentially limiting the evaluation of specificity and NPV estimation. This limitation is inherently related to the study population, as the pretest probability for disease based on the clinical scenario of elevated and/or rising PSA after curative‐intent therapy is very high. Our analysis is likely underpowered with respect to specificity and NPV determination; however, in the clinical scenario of biochemical recurrence, sensitivity and PPV are likely of greater import.

Interestingly, of 72 evaluable lesions identified, no lesions were located outside of the prostate bed, lymph nodes, or bone. This also likely relates to the study population with previous curative intent therapy and elevated and/or rising serum PSA.

Despite these limitations, the overall findings of this study add to the growing body of literature supporting the use of ^18^F‐PSMA‐1007 for lesion detection in recurrent prostate cancer. ^18^F‐PSMA‐1007 demonstrates reduced urine activity compared to other PSMA tracers (such as ^68^Ga‐PSMA‐11 or ^18^F‐DCFPyL) and may be uniquely suited to a high sensitivity for detecting disease in this patient population. Some studies have noted a uniquely high sensitivity for detecting bone lesions for this tracer, requiring unique interpretation criteria [12]. Further studies are needed to help refine the positivity criteria for lesion assessment, to better compare the properties and accuracy of different PSMA tracers in specific prostate cancer subpopulations, as well as to evaluate the effect on clinical outcomes for patients.

## 5. Conclusion

Overall, the findings of our study support the use of ^18^F‐PSMA‐1007 PET/CT over conventional imaging (CT chest, abdomen, pelvis, as well as a bone scan with SPECT/CT of the torso) in the evaluation of patients with BCR prostate cancer.

**Nomenclature**
PSMA: Prostate‐specific membrane antigen
PET: Positron emission tomography
CT: Computed tomography
MDP: Methylene diphosphonate
SPECT: Single‐photon emission computed tomography
BCR: Biochemically recurrent
PSA: Prostate‐specific antigen
eGFR: Estimated glomerular filtration rate
TP: True positive
FP: False positive
TN: True negative
FN: False negative
PPV: Positive predictive value
NPV: Negative predictive value

## Funding

A portion of this research was supported with funding from the Alberta Cancer Foundation and the Kaye Health Center Fund.

## Conflicts of Interest

The authors declare no conflicts of interest.

## Supporting Information

Additional supporting information can be found online in the Supporting Information section.

## Supporting information


**Supporting Information** Criteria for TP and FP Ffndings adapted from Lawhn‐Heath et al. [14].

## Data Availability

The data that support the findings of this study are available from the corresponding author upon reasonable request.
