# Supplementary material for: 18F‐PSMA‐1007 PET/CT Demonstrates Higher Accuracy for Biochemically Recurrent Prostate Cancer Compared to Contemporary Conventional Imaging
Source: Adv Urol. 2026 Jul 5;2026:8550174. doi: 10.1155/aiu/8550174 (PMC13333931; doi:10.1155/aiu/8550174)
Supplement: Supplementary file 1 — Supporting Information Criteria for TP and FP Ffndings adapted from Lawhn‐Heath et al. [14]. [file AIU-2026-8550174-s001.docx]

**Criteria for True Positive (TP) and False Positive (FP) Findings adapted from Lawhn-Heath *et al* (11)**

For a focus of abnormal ^18^F-PSMA-1007 uptake without histopathology assessment, the criteria for true positive and false positive findings were as follows:

**Lymph Nodes**

1. Interval systemic treatment

A. TP: > 50% decrease in PSA level and > 30% decrease in SAD at follow-up imaging, or > 50% increase in PSA level and >20% increase in SAD at follow-up imaging

B. FP: > 50% decrease in PSA level and > 20% increase in SAD at follow-up imaging

2. Interval local treatment without systemic treatment

A. TP: > 50% decrease in PSA level and < 20% increase and < 3 mm increase in SAD at follow-up imaging, or > 20% decrease in SAD regardless of PSA level

B. FP: PSA does not decrease > 50%, or > 20% increase in SAD at follow-up imaging

3. No interval treatment

A. TP: > 20% increase in SAD regardless of PSA level

B. FP: > 30% decrease in SAD regardless of PSA level

**Visceral lesions, prostate/prostate bed lesions**

1. Interval systemic treatment

A. TP: > 50% decrease in PSA and > 30% decrease in LAD at follow-up imaging, or > 50% increase in PSA level and > 30% increase in LAD

B. FP: > 50% decrease in PSA level and > 20% increase in LAD

2. Interval local treatment without systemic therapy

A. TP: > 50% decrease in PSA level or > 30% decrease in LAD

B. FP: < 50% decrease in PSA or > 20% increase in LAD

3. No interval treatment

A. TP: > 20% increase in LAD regardless of PSA

B. FP: > 30% decreased in LAD regardless of PSA

**Bone lesions**

A. TP: corresponding enhancing lesion on MRI, sclerotic lesion on CT, or focal uptake on bone scan on concomitant or follow-up imaging, or > 50% decrease in PSA level after targeted therapy

B. FP: PSA does not decrease > 50% after targeted therapy

PSA = prostate specific antigen

SAD = short axis diameter

LAD = long axis diameter
